# The Comparative Status Hypothesis: Inferences About Discrimination Vary Based on Identity Salience

**DOI:** 10.1177/01461672251347735

**Published:** 2025-06-30

**Authors:** Elizabeth A. Quinn-Jensen, Anh L. K. Tran, Sara E. Burke, Brenda Major, Zoe Liberman

**Affiliations:** 1 University of California Santa Barbara, USA; 2 Syracuse University, NY, USA

**Keywords:** discrimination, attributions, social status, race, gender

## Abstract

Past research examining when people label an outcome as discrimination has largely ignored contextual factors beyond the victim–perpetrator dyad. We contend that the person who benefits from the outcome—referred to here as competitor—also influences how likely someone is to be labeled a victim of discrimination. Specifically, we argue that because people hold multiple social identities, which vary in their perceived status, the identity of a target’s competitor can change how likely the same target is to be seen as a victim. In three studies, we show that White U.S. adults were more likely to infer that a target had faced discrimination when the target’s competitor highlighted a lower status aspect of the target’s identity. This pattern was seen for targets from multiple backgrounds, including Asian men, White women, and Asian women. These results highlight the importance of moving beyond the victim-perpetrator dyad when considering whether an outcome is seen as discrimination.

Whether or not a negative outcome is seen as discrimination often depends on the social identities of the people involved. For example, people may be more likely to infer discrimination when imagining a White woman who is denied a promotion in favor of a White man than when imagining the reverse (a White man being denied a promotion in favor of a White woman). Indeed, past research has highlighted the importance of identity status asymmetries, finding that prototypical acts of discrimination involve low-status “victims” and high-status “perpetrators” (e.g., Inman & Baron, 1996). However, such work has largely ignored the social identities of individuals outside of the perpetrator-victim dyad. Because many discrimination claims involve competition for resources (e.g., not winning a fellowship or gaining a promotion), another important social identity to consider is that of the competitor: The person who is chosen over the victim to receive a valued outcome. For example, if the same White woman were denied the same promotion in favor of a Black woman, people may not infer that the White woman faced discrimination. In this case, the identity of a target’s “competitor” changes which aspect of the target’s identity is highlighted: when a White woman target is competing with a White man, her gender may be highlighted, whereas when she is competing with a Black woman, her race may be highlighted. Perceivers may then use a comparison of the highlighted identity to decide whether the target was discriminated against. We term this idea the *Comparative Status Hypothesis*, which we test in three studies. Specifically, we suggest that when inferring whether someone has faced discrimination, perceivers consider whether the target (the potential “victim”) comes from a group that is perceived as lower or higher status than their competitor.

Interestingly, the Comparative Status Hypothesis moves beyond past research by widening the scope of which aspect(s) of identity are considered when perceivers are inferring discrimination. Previous research has primarily focused on one dimension of social identity at a time (e.g., highlighting just race or gender). However, everyone has multiple social identities, and many people hold some identities that are perceived as high(er) status and others that are perceived as low(er) status. Therefore, as in our example, the same person may be seen as more or less likely to have faced discrimination based on which of their identities is salient. According to the Comparative Status Hypothesis, when a competitor makes salient an aspect of a target’s identity that is seen as lower status than the competitor’s analogous identity, perceivers are more likely to infer that a target has faced discrimination.

## The Prototype Model of Attributions to Discrimination

The prototype model of attributions to discrimination asserts that people have prototypes—cognitive representations of the “average” member of a category (Fiske & Taylor, 1991; Rosch, 1978)—that they use to decide whether discrimination has occurred (Inman & Baron, 1996). These prototypes are learned by extracting common features from past instances of discrimination and often include attributes related to a person’s social identity (race, gender, appearance, behavior, etc.; Hogg, 1993; Medin, 1989). The more closely an outcome matches a discrimination prototype, the more likely that outcome is to be labeled as discrimination. Discrimination prototypes often focus on the social identities of the perpetrator, the target, and the relationship between the two (Lalljee et al., 1992; O’Brien et al., 2008; O’Brien & Merritt, 2022). Social identity prototypes are associated with some amount of perceived status. The perceived status that Americans ascribe to each group includes elements of actual status differences across groups as well as beliefs about status (which may not always track the actual status of individuals or groups). In the case of race, for example, a combination of historical belief systems and sociopolitical practices has cast racial minority groups as inferior (Acuña, 1981; Fredrickson, 2002; Molina, 2014). For instance, based on the history of enslavement of Black people, and discriminatory laws such as redlining, the racial hierarchy in the U.S. positions White Americans as the most dominant and advantaged group, and Black Americans as a significantly disadvantaged group (e.g., Sidanius & Pratto, 1999; Zou & Cheryan, 2017). Other racial groups are perceived as relatively intermediate. For example, Americans perceive Asian Americans as having more status than Black Americans (Bergsieker et al., 2010; O’Brien & Major, 2005). However, this assumption treats all Asian Americans as a monolith, when in reality differences in actual status exist within Asian American subgroups (Kuo et al., 2020), highlighting that perceived status does not always match the actual status of a group. In our work, we focus on (and measure) the perceived status of various social identities.

Because high-status groups have more influence and control over resources and, therefore, more opportunities to withhold resources from lower status group members (Sidanius & Pratto, 1999), people’s discrimination prototypes generally assume that perpetrators are members of high-status groups and targets are members of lower status groups. This is known as the status asymmetry hypothesis (Inman & Baron, 1996; Rodin et al., 1990). There is abundant support for this hypothesis across various types of social identity dimensions (race, gender, weight, age, sexual orientation; Baron et al., 1991; Carlsson & Sinclair, 2018; Krum & Corning, 2008; Marti et al., 2000; Morera et al., 2004; O’Brien et al., 2008, 2024; O’Brien & Merritt, 2022; Rodin et al., 1990; Simon et al., 2013). People are more likely to infer discrimination when the perpetrator is White, a man, or heterosexual versus when the target is a racial minority, a woman, or a sexual minority (Baron et al., 1991; Carlsson & Sinclair, 2018; Inman & Baron, 1996; Marti et al., 2000; Rodin et al., 1990).

## The Comparative Status Hypothesis

Past work on whether a negative outcome is seen as discrimination, however, has largely ignored an important contextual feature: the social identity of the victim’s most salient competitor. A victim may be more likely to be seen as having faced discrimination when the beneficiary of the potentially discriminatory act has a higher perceived social status than the victim. Because everyone has multiple social identities (race, gender, sexual orientation, etc.), and these identities come with varying levels of perceived status, the dimension of the victim’s identity that is made salient can change based on the identity of the competitor. For example, if an Asian man loses to an Asian woman, his *gender* may be made salient, and he might not be viewed as a victim of discrimination. But if an Asian man loses to a White man, then his *race* may be made salient, and he may be viewed as having faced discrimination since the competitor comes from a higher status racial group. Thus, simply knowing the social identities of the perpetrator (the person who makes the promotion decision) and the victim (who loses out) may not provide enough information to discern whether the outcome will be seen as discrimination.

The Comparative Status Hypothesis can perhaps best be understood by considering the lens-based model of intersectionality, which posits that an individual is not always evaluated based on all their social identities. Instead, this theory argues that people have multiple “lenses” through which they view others, and these lenses get selectively activated depending on the context (Petsko & Bodenhausen, 2020; Petsko et al., 2022). Specifically, perceivers can activate separate lenses when thinking about someone based on their race, gender, sexual orientation, and so on. For example, a Black man can be evaluated based on his race, his gender, or both identities, depending on the context. His Black identity may be more salient in some contexts (e.g., a Black Lives Matter protest), and his male identity may be more salient in others (e.g., a #MeToo protest). There is empirical evidence for this theory in the domain of stereotyping, where it has been shown that which dimension of identity is salient changes the stereotypes applied to an individual. When Asian women’s race is made salient, they are more likely to be stereotyped as “good at math,” whereas when gender is made salient, they are more likely to be stereotyped as “bad at math” (Pittinsky et al., 2006; Rattan et al., 2019).

The lens-based model of intersectionality also posits that whether a specific lens is activated over others may depend on *lens fit* (how closely a specific social identity lens explains a pattern of intergroup behavior typical in a context) and the *perceiver’s goals* (the desired outcome motivates the use of one social identity lens over another). For example, the pattern of behavior most typical in cases of discrimination is that someone who is of lower status is targeted. Thus, in cases where the competitor activates a lens that highlights a perceived lower status aspect of a target’s identity (compared to the competitor), the situation is more likely to fit a prototypical pattern of discrimination. A recent study provides suggestive evidence for the Comparative Status Hypothesis: Quinn-Jensen et al. (2024) found that when a bisexual woman lost out on a competitive funding opportunity to a heterosexual woman, she was more likely to be seen as having faced discrimination compared to when she lost out on the funding to a lesbian woman. Participants also perceived lesbian women as lower in status than bisexual women. Thus, the bisexual woman was more likely to be seen as a victim of discrimination when her competitor highlighted her lower perceived status. We expect that this pattern is indicative of a broader phenomenon that can apply to many types of social identity: When a competitor activates a lens through which the target’s lower status identity becomes salient, the target is more likely to be seen as having faced discrimination.

## Overview of Studies

Here, we provide evidence from three studies that support the Comparative Status Hypothesis. We show that White adults in the United States are more likely to say that a target faced discrimination when the competitor makes salient an identity for which the target is perceived to have *lower status* than the competitor. Because social norms discourage overt forms of prejudice against women and racial/ethnic minorities, and modern discrimination along these demographic dimensions is often covert (Bobo, 2001; Crandall et al., 2002), we focus on a scenario in which it is ambiguous whether the target faced discrimination. Specifically, participants read a vignette in which a target loses out on a competitive funding opportunity to another employee without being provided an explanation (adapted from Eliezer & Major, 2012; Quinn-Jensen et al., 2024).

Within each study, we held the identity of the target and the perpetrator constant to test the specific effect of the *competitor’s* identity. We tested our hypothesis with targets from three different social groups: an Asian man (Study 1), a White woman (Study 2), and an Asian woman (Study 3). Study 1 served as an initial test of the importance of competitor identity by only varying one dimension of identity: race. An Asian man lost out on funding to either a White man or a Black man. Because Asian Americans are perceived as having *more* status compared to other historically marginalized racial groups (e.g., Black and Hispanic people) but *less* status than White Americans (Zou & Cheryan, 2017), we predicted that the target would be more likely to be seen as a victim of discrimination when losing out to a higher status (White) competitor. Studies 2 and 3 tested whether activating an identity *lens* could change whether a target would be seen as having faced discrimination. In these studies, competitors differed from the target based on either racial identity or gender identity, allowing us to ask whether participants were viewing the scenario through a race-based lens or a gender-based lens when determining whether the target had faced discrimination. Specifically, we examined how the race and gender of the competitor changed whether a White woman (Study 2) or an Asian woman (Study 3) was seen as a target of discrimination.

For all studies, we report all measures and manipulations, how we determined our sample size, and the reasons for any exclusions. Only Study 3 was preregistered, but the data and codebooks for all studies can be found on OSF: https://osf.io/86phw/?view_only=ce60819112824f7080beb70ee46d2013

## Study 1

To begin investigating the Comparative Status Hypothesis, we asked whether an Asian American male target, a member of a racial group that is seen as holding an intermediate status, was more likely to be viewed as having faced discrimination after losing a higher status competitor (White man) as compared to a competitor perceived as lower status (Black man). We also included conditions in which the target was a Black American man competing against either a White man or an Asian man. We hypothesized that the Black man would be seen as having faced discrimination either way, since both White men and Asian men may be seen as relatively higher status.

## Method

### Participants

Since this was an initial test of the Comparative Status Hypothesis, we wanted to have sufficient power to test for a small effect size. An *a priori* power analysis using G*Power showed that 500 participants would give us 80% power to detect a small effect size of *f* = 0.13 with an alpha of .05 for an interaction between target race and competitor race (Faul et al., 2007). Because White people hold the most power and influence over resource distribution in the United States (U.S. Bureau of Labor Statistics, 2023), and because race may shape people’s perceptions of racial discrimination (e.g., O’Brien et al., 2024), we only recruited White participants (using the Prolific prescreen function) for this and subsequent studies.

Of our initial sample of 500 people, 69 participants racially identified as something other than White (despite having reported their race as White on the Prolific prescreen), leaving a final sample of 431 White participants (*M*_age_ = 42.74, *SD* = 14.44; 57% female, 41% male, 2% nonbinary, 1% gender identity not listed). Participants were paid $0.50 for participation. This study was approved by the university’s IRB.

### Procedure

This study used a 2 (apparent target race: Asian American vs. Black American) by 2 (competitor race: White American vs. different racial minority) between-subjects factorial design. Participants read about a target who worked at a law firm and applied for a law school funding opportunity. Depending on the condition, the target was depicted as either an Asian American man or a Black American man. In all cases, the target lost out to another employee, here referred to as the competitor. The competitor was randomly assigned to be depicted as either White or non-White. When the target was Asian, the non-White competitor was Black. When the target was Black, the non-White competitor was Asian. To manipulate the apparent race of the target and competitor, we used pictures from the Chicago Face Database (Ma et al., 2015). The database includes a norming survey conducted by Ma et al. (2015) in which each face was rated based on whether it was prototypical of a certain race. We chose to use faces that were highly prototypical of a single “apparent” race (at least a 5 on a scale from 1 = “*Not at all*” to 7 = “*Extremely*”). The apparent gender of each target was communicated via the names given to the target and competitors. Therefore, throughout the rest of the manuscript, when referencing the “race” or “gender” of the target, we are referring to his or her “apparent race/gender.” To select the final sample of 9 images (3 Black men, 3 Asian Men, and 3 White men), we chose stimuli that were also rated similarly on age (21–29-years old) and attractiveness (3.00–3.83 on a 1–7 scale), as these factors are associated with more success in the workplace (Nault et al., 2020; York, 2019). The final stimuli selected were also similar in perceived trustworthiness (3.12–4.47 on a 1–7 scale). For each participant, we drew stimuli randomly from the image sets corresponding to the condition. The norming data (Ma et al., 2015) for the images used is available in the Supplemental Material.

The vignette read:

“David^1^ and Michael both work as administrative assistants at a prestigious law firm. Every year the law firm offers law school funding to an especially promising administrative assistant. Steve is the boss and the most senior member of the firm, so he gets to choose who receives the funding. David and Michael each apply for the funding, along with several other employees. Both David and Michael take pride in their work and frequently spend long hours in the office, so they each think that they have a good chance of being selected. The following day David learns that Steve did not choose him to receive the law school funding. Instead, he chose Michael to receive the funding.”

After reading the vignette, participants answered questions about whether the target (David) was discriminated against.^2^ Then, they were told that the target filed a lawsuit alleging discrimination and were asked about their support for the target’s lawsuit. After, participants rated the perceived status of various racial groups. Participants then responded to two manipulation checks in which they were asked to identify the race of the target and competitor. Finally, participants reported their demographic information.

### Measures

#### Discrimination Variables

We measured whether participants viewed the target as having faced discrimination in a few ways. On one question, participants were asked to rate their agreement with the following statement: “[Target] was discriminated against based on his race,” with responses from 1 (“*strongly disagree*”) to 7 (“*strongly agree*”). Participants were also told that “there are many factors that could impact who was selected for the funding” and were asked to rate how much “each factor led to the decision not to fund [Target],” with higher scores indicating more agreement that the item contributed to the target not getting the funding. Two of the items related to discrimination: “[Target]’s race” and “Steve’s prejudice against [Target]’s race.” Participants also rated three other factors, which we call internal attributions: “[Target]’s qualifications,” “[Target]’s career ambitions,” and “[Target]’s work record.” All ratings were assessed on a scale from 1 (“*not at all*”) to 7 (“*very much*”). The three internal variables were averaged into a composite (α = .83), and the two discrimination attribution measures were composed with the overt discrimination question (called “discrimination claim,” α = .91).

#### Lawsuit Variables

Participants were told that “[Target] has filed a lawsuit against the company and against Steve alleging racial discrimination. The lawsuit asserts that [Target] was denied law school funding due to discrimination based on his race and seeks compensatory damages.”

We created two composite variables about participants’ judgments about the lawsuit. The first assessed how valid they viewed the lawsuit to be and was comprised of the following four items, all assessed on a scale from 1 (“*not at all*”) to 7 (“*very much*”) with higher scores indicating the target’s lawsuit was seen as more legitimate (α = .95): “Is [Target]’s lawsuit valid?,” “Do you support [Target] in filing this lawsuit?,” “Should [Target]’s lawsuit be taken seriously?,” and “Is [Target]’s case legitimate?” The second variable (α = .75) assessed views of how “a *real judg*e” and the participant would rule, from 1 (“*Definitely in favor of [Target]*”) to 7 (“*Definitely in favor of the firm and Steve*”).

#### Additional Variables

Next, participants were asked about their perceptions of the status of various racial groups. Specifically, they completed the MacArthur Scale of Subjective Social Status (Adler et al., 2000) in which they were shown a 10-step ladder and a short passage explaining that the numbers on the ladder represent a person’s status in America, with “the people who are the best off—those who have the most money, most education, best jobs, and those who have the authority to make important decisions [at the top]. At the bottom are the people who are the worst off—those who have the least money, least education, worst jobs, and have no authority to make important decisions.” Thus, higher numbers meant higher perceived social status. Participants placed each of the following groups on the ladder: Black Americans, Asian Americans, White Americans, and Hispanic Americans.

Finally, participants were asked to select the race/ethnicity of the target, the competitor, and the boss (Steve) from the following three options: “Asian,” “Black,” or “White.” For the target and the competitor, these questions were a manipulation check since the identity had been shown via pictures. The boss was not pictured in the task, so responses indicated participants’ expectations of his identity.

### Results

We conducted a between-subjects ANOVA with both conditions (target race: Asian vs. Black, competitor race: White vs. different racial minority) as predictors for all analyses unless specified otherwise. Because our main question concerns whether participants’ perceptions of discrimination vary based on the competitor’s identity (with participants being more likely to perceive discrimination when the competitor’s identity is higher status than the target) we are most interested in significant effects of competitor race (which could suggest more discrimination when the competitor is high status) and significant interactions between target race and competitor race (which could suggest that competitor race matters more for Asian targets, who hold intermediate status).

#### Discrimination Claim

**Figure 1.**
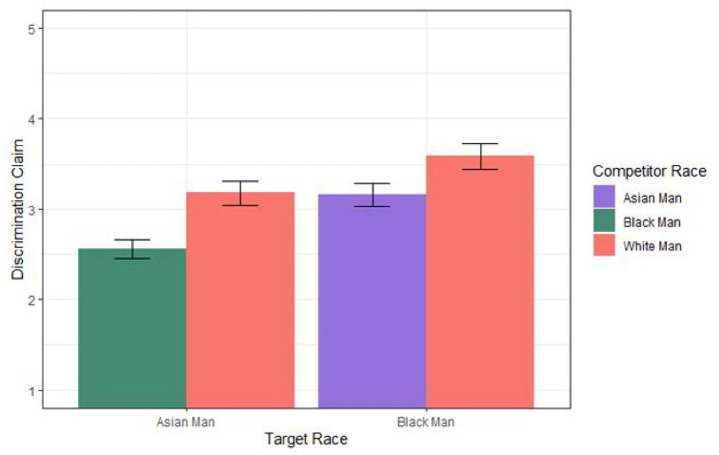
Discrimination claim measure results.

For the discrimination claim question, our ANOVA revealed a significant main effect of target race (*F*(1, 427) = 14.63 *p* < .001, η_р_^2^ = .03) and competitor race (*F*(1, 427) = 9.17, *p* = .003, η_р_^2^ = .02). See Figure 1. The effect of target race was due to participants perceiving Black targets (*M* = 3.25, *SD* = 1.65) as more likely to have faced discrimination than Asian targets (*M* = 2.68, *SD* = 1.45). In line with our primary hypothesis, the effect of competitor race was due to targets being perceived as significantly more likely to have faced racial discrimination when they lost out on the funding to the high-status White competitor (*M* = 3.19, *SD* = 1.69) compared to when they lost out to a racial minority competitor (*M* = 2.74, *SD* = 1.41). The interaction between target race and competitor race was not significant (*F*(1, 427) = 0.02, *p* = .892, η_р_^2^ = .00).

#### Lawsuit Legitimacy and Lawsuit Rulings

When rating the legitimacy of the target’s lawsuit, participants were most attentive to the target’s race: Our ANOVA revealed a main effect of target race (*F*(1, 427) = 4.93, *p* = .027, η_р_^2^ = .01) due to participants rating a lawsuit filed by the Black target (*M* = 3.70, *SD* = 1.79) as more legitimate than the lawsuit filed by the Asian target (*M* = 3.34, *SD* = 1.58, 95% CI [0.04, 0.68]). Competitor race (*F*(1, 427) = 2.43, *p* = .120, η_р_^2^ = .01) and the interaction between competitor and target race (*F*(1, 427) = 0.16, *p* = .693, η_р_^2^ = .01) were not significant. For questions on the lawsuit ruling, the ANOVA again revealed a significant main effect of target race (*F*(1, 427) = 5.05, *p* = .025, η_р_^2^ = .01) with participants reporting that they and a judge would be more likely to rule in favor of the Black target (*M* = 3.54, *SD* = 1.45) compared to the Asian target (*M* = 3.24, *SD* = 1.39, [0.04, 0.56]). However, in line with the Comparative Status Hypothesis, there was also a main effect of *competitor* race (*F*(1, 427) = 5.61, *p* = .018, η_р_^2^ = .01). Participants reported that rulings were more likely to be favorable for the target when the target lost to the (higher status) White competitor (*M* = 3.55, *SD* = 1.43) compared to when the target lost to a different racial minority competitor (*M* = 3.23, *SD* = 1.41, [0.06, 0.59]). Again, the interaction between competitor and target race was not significant (*F*(1, 427) = 0.27, *p* = .604, η_р_^2^ = .00).

#### Perceived Group Social Status

Replicating previous findings (see Zou & Cheryan, 2017), a repeated-measures linear ANOVA with a Greenhouse–Geisser correction revealed that participants expected members of different racial groups to hold different levels of status, *F*(2.22, 950.13) = 485.09, *p* < .001, η_р_^2^ = .53. Paired *t*-tests shows that participants perceived White Americans *(M* = 7.45, *SD* = 1.69) as having significantly more status than Asian Americans (*M* = 6.22, *SD* = 1.62; *t* = 13.12, *p* < .001, *d* = 0.74), Black Americans (*M* = 4.45, *SD* = 1.77; *t* = 26.72, *p* < .001, *d* =1.73), and Hispanic Americans (*M* = 4.47, *SD* = 1.64; *t* = 27.64, *p* < .001, *d* = 1.79). Asian Americans were also perceived as higher status than Black Americans (*t* = 18.15, *p* < .001, *d* = 1.04) and Hispanic Americans (*t* = 20.53, *p* < .001, *d* = 1.07). However, perceptions of status for Black Americans and Hispanic Americans did not differ (*t* = 0.33, *p* = .708, *d* = 0.01).

#### Manipulation Checks

Five participants incorrectly identified the target’s race, and 12 participants incorrectly identified the competitor’s race. Excluding participants who failed the manipulation check did not alter the substantive conclusions. Finally, most participants (96%) believed that Steve, the boss, was White.

### Discussion

Study 1 provided initial support for the Comparative Status Hypothesis. We found that, consistent with past work (Zou & Cheryan, 2017), participants viewed Asian Americans as having an intermediate status: lower than White Americans, but higher than Black Americans and Hispanic Americans. In line with this perception, the race of both the target *and* the race of the competitor affected participants’ judgments of discrimination. That is, Asian targets, who were viewed as higher status than Black targets, were seen as less likely to have faced discrimination. This finding is in line with past work suggesting that victims who are more prototypically low status are more likely to be seen as having faced discrimination (Major et al., 2002). Additionally, in line with the Comparative Status Hypothesis, targets who lost out to a competitor who was high in perceived status (a White man) were seen as more likely to have faced discrimination. That is, even when the identity of the perpetrator and target remained constant, the identity of the competitor changed how likely a person was to be seen as having faced discrimination. Interestingly, the identity of the competitor had the same effect for both Asian and Black targets.

It may initially seem like the Comparative Status Hypothesis would lead to a significant interaction between target and competitor race. That is, because a Black target is from a group with lower perceived status than either a White or Asian competitor, competitor race could have mattered more for an Asian target than a Black target. However, we believe that the observed findings are still in line with the Comparative Status Hypothesis. That is, because the Asian target has *lower* perceived status than the White target, the Black target is seen as even more likely to have faced discrimination when losing out to a White competitor. Ratings of discrimination were *high* even when the Black target’s competitor was Asian, suggesting that participants did see a Black target as having faced discrimination with either competitor. Indeed, participants rated a Black target who lost out to an Asian competitor to be as likely as an Asian target who lost out to a White competitor to have faced discrimination (*M*_ratings_ = 3.01 and 2.89, respectively). In other words, the only case in which ratings of discrimination were “low” was when an Asian target lost out to a Black competitor.

## Study 2

Study 2 had two primary aims. First, we sought to conceptually replicate our findings from Study 1 in support of the Comparative Status Hypothesis: We again expected participants to be more likely to infer that a target faced discrimination when losing out to a higher status competitor. Second, we intended to test the lens-based theory more fully by varying the race and gender of the competitor. Specifically, we presented all participants with a White woman who lost out on a funding opportunity, but we varied whether the competitor highlighted her racial identity or her gender identity. To do so, we presented cases in which the funding was awarded to a Black woman (highlighting the White female target’s racial identity and relatively *high* status) or to a White man (highlighting the target’s gender identity and relatively *low* status).

We hypothesized that the target would be more likely to be seen as a victim of discrimination when she lost to the White man. We also included a third condition in which the competitor’s social identity was unstated. This condition may provide an estimate of how much general discrimination participants perceive the target group to face. Also, we expected that participants would assume that an unidentified competitor would be a White man. Just as White men are viewed as prototypical perpetrators of discrimination (Major et al., 2002), when a target alleges discrimination people may infer that the beneficiary was also a White man. Thus, we were interested in (a) whether participants would view the target as having faced more discrimination when losing to a White man compared to a Black woman and (b) whether views on likely discrimination would be similar when the target lost to a White man and to an unstated competitor.

## Method

### Participants

We recruited 600 White Americans from Prolific to have 80% power to detect an effect size of 0.13 with an alpha of .05 for the main effect of competitor social identity. We excluded 10 participants for identifying as something other than White on the survey, leaving us with a final sample of 590 (*M*_age_ = 41.37, *SD* = 14.06; 47% female, 51% male, 2% nonbinary, and 1% gender identity not listed). Participants were paid $0.50 for their participation, and this study was approved by the university’s IRB.

### Procedure and Measures

We used a 3 condition between-subjects design in which a White woman^3^ lost to either a Black woman competitor, a White man competitor, or a competitor of unstated social identity. Race and gender were again manipulated using images taken from the Chicago Face Database, and images were matched on perceived age, attractiveness, racial prototypicality, and trustworthiness (see Supplemental Material) in the context of the same law firm scenario as Study 1. Participants answered the same questions from Study 1 regarding internal-related attributions about why the target did not get the funding, as well as discrimination-related attributions. Participants also answered direct questions about whether the target had faced discrimination. But, rather than one question, we included separate questions about whether the target was discriminated against based on her race and based on her gender (to account for the different competitors, see full wording below). Finally, participants completed a measure comparing the perceived status of the target and competitor’s social identities.

#### Gender Discrimination

Participants were asked, “Do you believe [Target] was discriminated against based on her gender?” and responses were rated from 1 (“*strongly disagree*”) to 7 (“*strongly agree*”).

#### Race Discrimination

Participants were also asked, “Do you believe [Target] was discriminated against based on her race?” and responses were rated from 1 (“*strongly disagree*”) to 7 (“*strongly agree*”).

#### Perceived Status Difference

Participants were asked “Which of the following two groups do you think has higher status in our society” on a scale from 1 (*White women have higher status than* [*White men/Black women*]) to 7 ([*White men/Black women*] *have higher status than White women*). The groups in brackets differed by condition. Higher scores indicated that participants believed that the competitor had higher status than the target.

### Results

We conducted a one-way ANOVA with condition (competitor identity: White man vs. Black woman vs. unspecified) as the predictor for all analyses.^4^ For all significant main effects, we followed up with pairwise *t*-tests arising from individual indicator variables in the same model (unadjusted contrasts).^5^ Finding a significant effect of competitor identity on discrimination-based questions would provide support for the Comparative Status Hypothesis.

#### Discrimination Claim

Consistent with the Comparative Status Hypothesis, the effect of competitors’ identity was significant for the discrimination claim measure (*F*(2, 587) = 21.45, *p* < .001, η_р_^2^ = .07. Follow-up tests revealed that all conditions significantly differed from each other. Participants were more likely to infer discrimination when the target lost to the White man (*M* = 3.36 *SD* = 1.40) compared to the Black woman (*M* = 2.50, *SD* = 1.28, *p* < .001, 95% CI [0.60, 1.12], *d* = 0.64) or when the competitor was unstated (*M* = 3.03, *SD* = 1.25, *p* = .012, [0.08, 0.59], *d* = 0.25). People were also more likely to infer discrimination when the competitor was unstated compared to when the competitor was a Black woman (*p* < .001, [−0.78, −0.26], *d* = 0.43).

#### Gender and Race Discrimination

**Figure 2:**
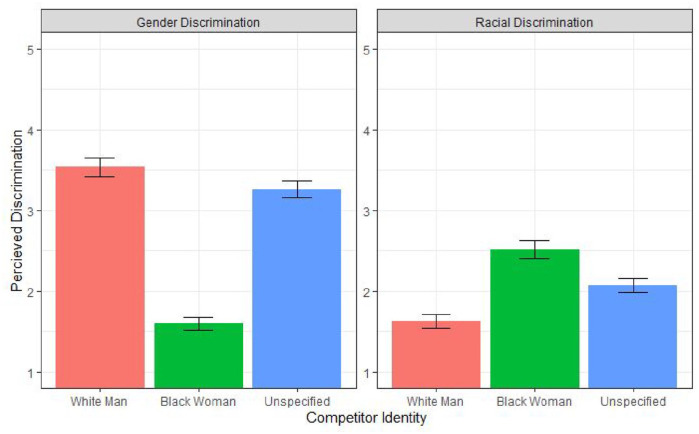
Study 2 gender and racial discrimination measure results.

Responses to the questions on gender and race discrimination revealed that participants were indeed paying attention to the expected dimension of identity when inferring discrimination. That is, there was a significant effect of competitor identity for both the gender discrimination measure, *F*(2, 587) = 103.34, *p* < .001, η_р_^2^ = .26, and the race discrimination measure *F*(2, 587) = 22.10, *p* < .001, η_р_^2^ = .07. See Figure 2. The target was more likely to be seen as a victim of gender discrimination when she lost out on the funding to a White man (*M* = 3.53, *SD* = 1.68) or when the competitor was unstated (*M* = 3.26, *SD* = 1.46) compared to a Black woman (*M* = 1.60, *SD* = 1.14; White man vs. Black woman: *p* < .001, 95% CI [1.65, 2.22], *d* = 1.34; Black woman vs. unstated: *p* < .001, [−1.95, −1.37], *d* = 1.27). For gender-based discrimination, there was no significant difference between the White man and the unstated competitor conditions, *p* = .058, [−0.01, 0.56], *d* = 0.17. In contrast, for race-based discrimination, all conditions differed from each other (*ps* < .001, *d*s > 0.32): the target was least likely to be seen as a victim of race-based discrimination when she lost out on the funding to a White man (*M* = 1.63, *SD* = 1.20), followed by when she lost to a competitor of unstated identity (*M* = 2.07, *SD* = 1.22), and finally by when the competitor was a Black woman (*M* = 2.52, *SD* = 1.54).

#### Lawsuit Legitimacy and Lawsuit Rulings

The competitor also had a significant effect on ratings of lawsuit legitimacy (*F*(2, 587) = 10.17, *p* < .001, η_р_^2^ = .03) and lawsuit rulings (*F*(2, 587) = 14.97, *p* < .001, η_р_^2^ = .05). The target’s lawsuit was perceived as more legitimate when she lost out on the funding to a White man (*M* = 3.67, *SD* = 1.76) or the competitor was unstated (*M* = 3.42, *SD* = 1.47) compared to a Black woman (*M* = 2.94, *SD* = 1.65; White man vs. Black woman: *p* < .001, 95% CI [0.41, 1.05], *d* = 0.4; Black woman vs. unstated: *p* = .004, [−0.81, −0.16], *d* = 0.31). However, there was no significant difference between the White man and unstated conditions, *p* = .140, [−0.08, 0.57], *d* = 0.15.

A similar pattern emerged for lawsuit rulings (*F*(2, 587) = 14.97, *p* < .001, η_р_^2^ = .05): Participants said that they and a real judge would be more likely to rule in favor of the lawsuit (α = .814) when the target lost out to a White man (*M* = 3.33, *SD* = 1.52) or unstated (*M* = 3.25, *SD* = 1.27) compared to a Black woman (*M* = 2.63, *SD* = 1.34; White man vs. Black woman: *p* < .001, 95% CI [0.42, 0.97], *d* = 0.49; Black woman vs. unstated: *p* < .001, [−0.89, −0.34], *d* = 0.47). However, there was no significant difference in lawsuit ruling judgments between the White man and unstated conditions, *p* = .577, [−0.20, 0.35], *d* = 0.06.

#### Perceived Status Difference

Results of two separate linear regressions with perceived status as the predictor variable and the discrimination claim measure as the outcome variable revealed results consistent with the Comparative Status Hypothesis. Specifically, participants who perceived a greater status differential between Black women and White women (with Black women having *less* perceived status) were less likely to report that a White woman who lost out to a Black woman had experienced discrimination (β = −.28, *SE* = 0.06, *p* < .001). Additionally, participants who perceived a greater status differential between White men and White women (with White having *less* perceived status) were more likely to report that a White woman who lost out to a White man had experienced discrimination (β = .29, *SE* = 0.06, *p* < .001).

### Discussion

Results of Study 2 again provided support for the Comparative Status Hypothesis. Participants were less likely to infer that a White woman had faced discrimination and viewed her lawsuit as less legitimate when she lost to a Black woman as opposed to either a White man or a competitor of unstated identity. That is, when the competitor highlighted the target’s relatively *higher* (racial) status, participants were less likely to report that she had faced discrimination. This interpretation is supported by our race and gender discrimination measures, which showed that participants were paying attention to the relevant social identity dimensions in each comparison: They reported that discrimination based on a particular dimension was more likely when the target and competitor differed on that dimension. So, when asked about gender discrimination, participants reported that a White woman who lost to a man was more likely to have faced discrimination than a White woman who lost to another (Black) woman. And, when asked about race discrimination, participants reported that it was more likely when the victim and competitor belonged to different groups (the White woman lost to a Black woman) than when they were members of the same group (both White).^6^ Participants’ responses to the status difference measure provided additional support to the Comparative Status Hypothesis. We show that the more participants perceived the target’s social identity to be of lower status than the competitor, the more likely they were to say that the target faced discrimination.

Interestingly, the results of the unstated competitor condition suggest that people may not always infer that the competitor is a White man. If they had, the unstated competitor condition would not differ from the condition in which the target lost to a White man. Although this was true for some measures (e.g., Steve’s prejudice, lawsuit legitimacy), other measures revealed that participants were more likely to infer discrimination when the competitor was clearly stated to be a (higher status) White man than when the competitor’s identity was not mentioned (e.g., the discrimination claim, racial discrimination). However, for all dependent variables, participants were *less likely* to infer discrimination when the target lost out to a Black woman compared to the unstated competitor. Thus, participants may assume that the competitor is of relatively higher status than the White female target (but not always a White man). It is possible that this assumption helps participants make sense of the target’s lawsuit alleging discrimination, which may indicate that at least the target sees the competitor as a higher status.

## Study 3

Study 3 sought to conceptually replicate findings from Study 2 with a target who could more plausibly experience discrimination based on either their race or gender: an Asian woman. We presented participants with an Asian woman who was denied funding and again varied whether the competitor highlighted her racial identity or her gender identity. That is, the target lost out on the founding to a White woman (highlighting her race and relative low status), an Asian man (highlighting her gender and relative low status), or a Black woman (again highlighting her race, but implying relative *higher* status). We hypothesized that an Asian woman would be more likely to be seen as a target of discrimination when she lost to an Asian man or a White woman as opposed to a Black woman.

## Method

### Participants

We recruited 600 White participants from Prolific (prolific.com) for this preregistered study. Results of an a priori power analysis showed that recruiting 567 participants would provide 90% power with an alpha of .05 to detect the smallest effect size (*f* = 0.15) observed in Study 2. However, we oversampled to account for possible exclusions. We excluded 11 participants for identifying as something other than White on the survey, leaving us with a final sample of 589 (*M*_age_ = 42.01, *SD* = 14.10; 49% female, 50% male, and 1% nonbinary). Participants were paid $0.50 for their participation, and this study was approved by the university’s IRB. Preregistration can be found on OSF (https://osf.io/zub7p/?view_only=9cbc1e95716e48279b2f0ffe06733f1b).

### Procedure and Measures

This study was a three-condition (competitor identity: Asian man vs. Black Woman vs. White woman) between-subjects factorial design. We used the same manipulation and measures from Study 2,^7^ with the addition of some wording changes to the perceived status difference measure (see below).

#### Perceived Status Difference

Participants were again asked “Which of the following two groups do you think has higher status in our society” on a scale from 1 (*Asian women have higher status than* [*Asian men/White women/Black women*]) to 7 ([*Asian men/White women/Black women*] *have higher status than Asian women*). Higher scores indicated that participants believed that the competitor had higher status than the target.

### Results

We conducted a one-way factorial ANOVA with condition (competitor identity: Asian man vs. Black woman vs. unspecified) as the predictor for all analyses. For all significant main effects of condition, we followed up with pairwise *t*-tests arising from individual indicator variables in the same model (unadjusted contrasts).^8^

#### Discrimination Claim

Consistent with the Comparative Status Hypothesis, there was a significant effect of the competitor’s identity on the discrimination claim measure (*F*(2, 586) = 14.68, *p* < .001, η_р_^2^ = .05). Participants were less likely to infer discrimination when the target lost to a Black woman (*M* = 2.42, *SD* = 1.42) compared to either an Asian man (*M* = 2.97, *SD* = 1.30; *p* < .001, 95% CI [−0.82, −0.27], *d* = 0.40) or a White woman (*M* = 3.14, *SD* = 1.42; *p* < .001, [−1.00, −0.45], *d* = 0.51). However, there was no significant difference between the conditions where the target lost to an Asian man or a White woman (*p* = .211, [−0.45, 0.10], *d* = 0.12).

#### Gender and Race Discrimination

**Figure 3.**
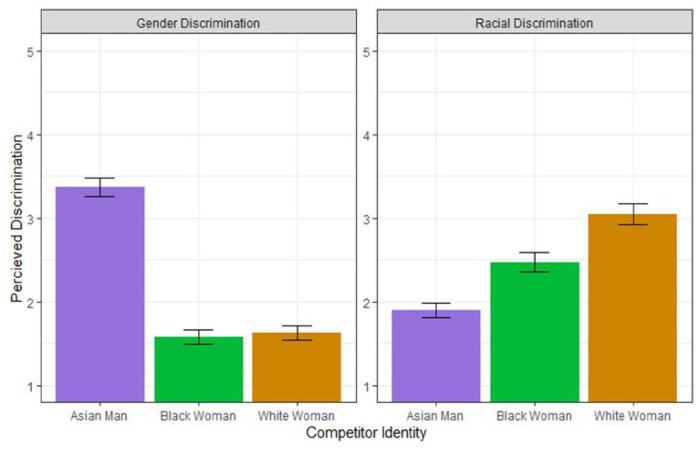
Study 3 gender and racial discrimination measure results.

Competitor identity significantly impacted responses on both the gender (*F*(2, 586) = 114.22, *p* < .001, η_р_^2^ = .28) and race discrimination (*F*(2, 586) = 27.70, *p* < .001, η_р_^2^ = .09) measures, showing that participants were focusing on the expected dimension of identity. See Figure 3. Participants were more likely to report that the target faced gender discrimination when the gender of the target and the competitor differed (when she lost to an Asian man, *M* = 3.37, *SD* = 1.57) compared to when the competitor was also a woman (White woman: *M* = 1.63, *SD* = 1.21; Black woman: *M* = 1.57, *SD* = 1.19; Asian man vs. White woman: *p* < .001, 95% CI [1.48, 2.00], *d* = 1.24; Asian man vs. Black woman: *p* < .001, [1.53, 2.06], *d* = 1.03). However, there were no significant differences in reported gender discrimination when the competitor was also a woman, regardless of the competitor’s race (White woman vs. Black woman: *p* = .695, [−0.21, 0.32], *d* = 0.04). For race-based discrimination, all conditions significantly differed from each other. The target was most likely to be seen as a victim of race-based discrimination when she lost out to a White woman (White woman, *M* = 3.05, *SD* = 1.68) compared to when she lost out to an Asian man (*M* = 1.90, *SD* = 1.21, *p* < .001, [0.85, 1.45], *d* = 0.79) or a Black woman (*M* = 2.47, *SD* = 1.65, *p* < .001, [0.28, 0.88], *d* = 0.35). The target was also more likely to be seen as a victim of race-based discrimination when she lost out to the Black woman compared to the Asian Man (*p* < .001, [0.27, 0.87], *d* = 0.39).

#### Lawsuit Legitimacy and Lawsuit Rulings

The competitor identity had a significant effect on ratings of lawsuit legitimacy (*F*(2, 586) = 5.93, *p* = .003, η_р_^2^ = .02) and lawsuit rulings (*F*(2, 586) = 4.35, *p* = .013, η_р_^2^ = .02). The target’s lawsuit was perceived as more legitimate when she lost to an Asian man (*M* = 3.44, *SD* = 1.62) or a White woman (*M* = 3.45, *SD* = 1.71) compared to a Black woman (*M* = 2.94, *SD* = 1.69; Asian man vs. Black woman: *p* = .003, [0.17, 0.83], *d* = 0.30; White woman vs. Black woman: *p* = .003, [0.18, 0.84], *d* = 0.30). However, there was no significant difference between the Asian man and White woman conditions (*p* = .941, [−0.35, 0.32], *d* = 0.01). A similar pattern was found for lawsuit rulings: participants said that they and a real judge would be more likely to rule in favor of the target when she lost to an Asian man (*M* = 2.98, *SD* = 1.43) or White woman (*M* = 3.15, *SD* = 1.49) compared to a Black woman (*M* = 2.74, *SD* = 1.40; Asian man vs. Black woman: *p* = .030, [0.03, 0.60], *d* = 0.23; White woman vs. Black woman: *p* = .005, [0.12, 0.68], *d* = 0.28). No significant difference emerged between the Asian man and White woman conditions (*p* = .533, [−0.37, 0.19], *d* = 0.06).

#### Perceived Status Difference

We again ran three separate linear regressions with perceived status difference as the predictor variable and the discrimination claim measure as the outcome variable. The effect of perceived status was strongest for participants’ perceptions of the status of White women versus Asian woman. That is, participants who perceived a greater status differential between White women and Asian women (with Asian women having *less* perceived status) were significantly more likely to report that an Asian woman who lost out to a White woman had experienced discrimination (β = .20, *SE* = 0.07, *p* = .006). These effects were not significant for other comparisons. Participants who perceived a greater status differential between Asian men and Asian women (with Asian women having *less* perceived status) were only marginally more likely to report that an Asian woman who lost out to an Asian man had experienced discrimination (β = .12, *SE* = 0.07, *p* = .082). Finally, perceptions of Black and Asian women’s status differential did not predict participants’ reports of discrimination when an Asian woman lost out to a Black woman (β = .09, *SE* = 0.07, *p* = .197).

### Discussion

Results of Study 3 again offered support for the Comparative Status Hypothesis, demonstrating that the same target who lost out on the same opportunity (which was awarded by the same “perpetrator”) was more likely to be seen as a victim of discrimination when the competitor was perceived to be relatively higher status than the target (on the relevant dimension). That is, when an Asian woman lost out to an Asian man, she was viewed through the lens of her gender and seen as a target of discrimination. But, when the Asian woman lost out to another woman, she was viewed through the lens of her race and more likely to be seen as facing discrimination when she lost to a White woman (competitor was higher status) than when she lost to a Black woman (competitor was likely seen as lower status). This was supported by our race and gender discrimination measures: Participants reported that discrimination was more likely when the target and the competitor differed on a social identity dimension, and particularly when the target was lower status than the competitor. This was also partially supported by our measure of perceived status differences: The greater perceived status difference between Asian women and White woman, the more likely participants were to say that an Asian woman who lost out to a White woman had been a victim of discrimination. Interestingly, perceptions of status differences did not significantly predict participants’ judgments when inferring discrimination of an Asian woman who lost out to a Black woman or an Asian man. One potential explanation for this finding is that the competitor’s social identity may highlight different dimensions of status, and our measure was not nuanced enough to pick up on these differences for all comparisons. For example, it has been proposed that perceived status consists of multiple dimensions (e.g., wealth, dominance, decision-making power, & prestige; Enright et al., 2020). It is possible that our status differential measure was capturing one (or more) of these dimensions more than others.

## General Discussion

Three studies provided evidence in support of the Comparative Status Hypothesis, demonstrating that whether a target is seen as having faced discrimination depends not only on their identity, but also on the identity of their competitor. When a target loses out to a relatively higher status competitor, they are more likely to be seen as a victim of discrimination than when the same target loses out to a relatively lower status competitor. This was true for targets with different social identities, including an Asian man, a White woman, and an Asian woman, suggesting the phenomenon is not specific to one group of people but may apply to any target that holds identities that may be seen as higher status than some competitors and lower status than other competitors. Our work conceptually replicates the findings of Quinn-Jensen et al. (2024), who demonstrated that a bisexual woman was more likely to be seen as having faced discrimination when losing out to a heterosexual competitor (higher status) than a lesbian competitor (perceived lower status). Thus, although past research on when people make attributions to discrimination has focused almost exclusively on the relationship between the perpetrator and victim, this research demonstrates that it is essential to consider the larger social context and other identities (including that of the competitor). The current research broadens our understanding of the prototype model of attributions to discrimination (Inman & Baron, 1996) by demonstrating that people use additional contextual information to assess whether a person is a more prototypical or less prototypical victim of discrimination. In doing so, this research opens the door for future research to consider how other contextual factors affect victim prototypicality and attributions to discrimination (see Limitations and Future Directions for suggestions).

This research also demonstrates the importance of considering more than one social identity dimension when evaluating whether people will infer that a negative outcome is discrimination. Past work on discrimination attributions has focused primarily on one dimension of social identity at a time, evaluating perpetrators and victims who vary only on *either* their gender, race, or sexual orientation (e.g., Marti et al., 2000; Morera et al., 2004; O’Brien et al., 2008, 2024; O’Brien & Merritt, 2022). However, people have multiple social identities, and which social identity “lens” is activated can depend on the context. In line with the lens-based model of intersectionality (Petsko & Bodenhausen, 2020; Petsko et al., 2022), a competitor changed how likely a person was to be seen as a target of discrimination by making salient different social identities. For example, when a target and competitor differed in gender, gender identity was salient, but when a target and competitor differed in race, racial identity was more salient. As evidenced by our findings, when a competitor made salient the target’s lower status identity, perceivers were more likely to infer that the target had faced discrimination.

Thus, the lens-based model of intersectionality can be applied to understand person perception outside of the domain of stereotypes, the context in which it has been previously investigated (Petsko & Bodenhausen, 2020; Petsko et al., 2022). For example, both the Comparative Status Hypothesis and the lens-based model could be used to study how voters perceive and respond to political candidates. Specifically, how do people perceive the leadership skills of a White female political candidate when her competitor is a White man compared to a Black woman? A White male competitor may make the candidate’s gender salient, highlighting the stereotype of women as less effective leaders (Eagly & Karau, 2002). However, a Black female competitor could instead make the candidate’s race salient, highlighting the stereotype of White people as competent (Fiske et al., 2018).

Finally, this research also suggests that the role a competitor plays in whether an outcome is seen as discrimination may have real-world implications. That is, when the target’s status was relatively higher than the competitor’s status, participants perceived the target’s lawsuit as less legitimate and supported the lawsuit less. This is consistent with past research showing that people view a claimant’s discrimination lawsuit as less legitimate when the victim is perceived as less prototypical of discrimination targets (Goh et al., 2021; Quinn-Jensen et al., 2024).

### Limitations and Future Directions

This research focused on White adult participants because they hold the most social power in the United States (U.S. Bureau of Labor Statistics, 2023). However, it is important for future research to understand whether perceivers from other racial and ethnic backgrounds similarly use the competitor’s social identity to infer whether a target has faced discrimination. Past research has shown that people from different backgrounds in the United States typically hold similar discrimination prototypes (e.g., men and women both perceive women as more prototypical victims of discrimination than men; O’Brien et al., 2008), suggesting that the perceiver’s background may not usually determine inferences about discrimination that are based on whether a target fits the prototype of being a “victim.”

However, it is also possible that even if people have shared prototypes of a “typical” victim, a perceiver’s social identity could play a role in their judgments of discrimination. In that case, racial minority participants may respond differently when the competitor shares their racial identity versus belonging to a different minority group. For example, perceivers may be more likely to see discrimination when the “victim” matches their identity. In this case, a Black woman might be more likely than a Hispanic woman to report that a Black woman losing out to a Hispanic woman had faced discrimination (whereas the Hispanic woman might be more likely than the Black woman to report discrimination when a Hispanic woman lost out to a Black competitor). Indeed, a recent study found that whereas White and Black Americans were more likely to infer discrimination after a Black candidate failed to get a job compared to an Asian candidate, Asian participants rated both candidates as equally likely to have faced discrimination (O’Brien et al., 2024). That is, sharing in-group status with the potential victim was associated with more perceptions of bias. It is also possible that racial minority participants would use competitor identity in a manner similar to White participants but that they would have a lower bar for what “counts” as discrimination. If so, they may be more inclined to label ambiguous acts as discrimination (e.g., Johnson et al., 2003; Monteith & Spicer, 2000; Sommers & Ellsworth, 2000). For example, Sommers and Norton (2006) found that non-White participants were more likely than White participants to label subtle acts of bias as racism. Therefore, future research should recruit diverse samples and more directly test the role of perceiving each party (the target, the competitor, and even the perpetrator) as an in-group member.

Because we recruited only White participants, our findings could also be interpreted through the lens of lay theories of prejudice and discrimination as proposed by Sommers and Norton (2006). Sommers and Norton (2006) found that, compared to racial minorities, White people’s conceptualization of racism is more in line with “old-fashioned” racism (e.g., making jokes about Black people) than with modern, subtle racism (e.g., hiring a White candidate over a Black candidate for a job). However, White people who were more internally motivated to respond to prejudice and who scored higher on the Modern Racism Scale were more likely to label subtle racism as such. Therefore, White people may be motivated by different factors than racial minorities when labeling ambiguous acts as discrimination. Future research should explore these different motivations.

Relatedly, although we explored participants’ inferences about discrimination against Black men and Asian men (Study 1), White women (Study 2), and Asian women (Study 3), there are many other social identities that can be tested. Also, although we did not explicitly reveal the perpetrator’s racial identity, most participants believed that Steve, the boss, was White (see results for Study 1). Future work may investigate how the perpetrator’s identity (when stated) interacts with that of the target and competitor in participants’ judgments of discrimination. For example, participants may expect perpetrators to favor their own groups. Past research has shown that under certain circumstances both low- and high-status groups will demonstrate in-group favoritism during resource allocation tasks (Brewer, 1979; Mullen et al., 1992; Rubin et al., 2014). If perceivers do believe that perpetrators will show in-group favoritism, explicitly stating that a person from a historically marginalized racial background made the decision about who was rewarded could impact the likelihood of the action being labeled as discrimination. Or, participants may expect that historically marginalized groups are generally less likely to discriminate. To test these questions, researchers could ask whether a White woman who lost out to a Black woman was seen as having faced more discrimination, less discrimination, or the same amount when the boss making the decision (perpetrator) was White versus Black.

The Comparative Status Hypothesis is based on the importance of taking the broader context (in this case, the competitor’s identity) into account when testing inferences of discrimination. There are many other interesting aspects of context to explore. For example, researchers may be interested in understanding contexts that vary in terms of which group is perceived to be high status. The current studies were set in law firms, which are numerically dominated by upper-class, White men (86%; American Bar Association, 2020). Thus, the group in power in the context (White men) was the same as that in society at large. However, it is possible that there are other contexts in which the numerical majority or high-status group may differ from the typical power structure in the United States (e.g., there are more women than men serving as primary and secondary teachers or in nursing professions; women’s gymnastics is more high status than men’s gymnastics), and that expectations about who is a victim would vary in these cases.

Lastly, to avoid explicitly labeling each target and competitor’s race and gender (which we thought would draw too much attention to our research questions), we used photos to manipulate the race and gender of the target and competitor. For this reason, we were careful to note that we were actually manipulating the target’s apparent race. However, using images to manipulate apparent race may emphasize or even exaggerate participants’ existing beliefs about differences between racial groups (Martinez, 2023). For example, selecting images that were rated as highly prototypical of a specific racial group could reduce the likelihood that perceivers spontaneously think about diversity within these racial groups. Further, other works suggest that the same face may be racially categorized differently depending on contextual factors (Freeman et al., 2011; Nicolas & Skinner, 2017). We recommend that future research consider other strategies for investigating the role of race, including using data-driven methods to determine which specific features are impacting judgments (see Martinez, 2023).

## Conclusion

In the past decade, there has been a proliferation of research on when people see a negative outcome as discrimination. Most have focused on the social identities of the perpetrator and target. Our work highlights that it is critical to consider the social identity of the competitor when determining whether a negative outcome is viewed as discrimination.

## Supplemental Material

sj-docx-1-psp-10.1177_01461672251347735 – Supplemental material for The Comparative Status Hypothesis: Inferences About Discrimination Vary Based on Identity Salience Supplemental material, sj-docx-1-psp-10.1177_01461672251347735 for The Comparative Status Hypothesis: Inferences About Discrimination Vary Based on Identity Salience by Elizabeth A. Quinn-Jensen, Anh L. K. Tran, Sara E. Burke, Brenda Major and Zoe Liberman in Personality and Social Psychology Bulletin

## Notes

1. The name of the target was randomized: David, John, or James.

2. In addition to our main measures of discrimination attribution, we also included measures of internal attributions for why the target may not have received the funding (e.g., work record, career ambitions, etc.). Such internal attributions have been either negatively related (Crocker & Major, 1989; Major et al., 2002), or unrelated (O’Brien & Merritt, 2022; Schmitt & Branscombe, 2002) to discrimination attributions. See Supplemental Material.

3. We again randomized the name of the target: Michelle, Sarah, or Emily. The competitor’s name was always Julie.

4. Sixteen participants incorrectly identified the competitor’s race, and excluding participants who failed the manipulation check did not influence the substantive conclusions.

5. We report the effect of our manipulation on the discrimination claim and four other variables of interest. Some readers may worry that the inclusion of additional variables (e.g., lawsuit legitimacy) inflates the false positive rate of our testing procedure. Therefore, we subjected the set of 15 *p*-values from the pairwise condition comparisons for all five response variables to a Holm–Bonferroni correction procedure. The adjusted threshold was 0.017, and all comparisons reported as significant met this threshold anyway.

6. Although participants reported *more* race-based discrimination when a White woman lost to a Black woman, it is worth noting that their general agreement with the question was low, indicating that they did not tend to think that the White woman was experiencing “reverse discrimination” but rather just note that race could play more of a role when the victim and competitor come from different (rather than the same) racial groups.

7. We added additional manipulation check questions to see if participants were noticing both the gender and race of the target and competitor. See Supplemental Material. As with Study 2, we again randomized the name of the target: Michelle, Sarah, or Emily. The competitor’s name was always Julie.

8. Twenty-seven participants incorrectly identified the target’s gender or race, while twenty-eight participants incorrectly identified the competitor’s gender or race. Excluding participants who failed the manipulation check did not alter the conceptual conclusions. As in study 2, some readers may worry that the inclusion of additional variables (e.g., lawsuit legitimacy) inflates the false positive rate of our testing procedure. Therefore, we again subjected the set of 15 *p*-values from the pairwise condition comparisons for all five response variables to a Holm–Bonferroni correction procedure. The adjusted threshold was 0.010, and all comparisons reported as significant met this threshold except for the Asian man versus Black woman comparison for the lawsuit rulings.
