# Supplementary material for: The Comparative Status Hypothesis: Inferences About Discrimination Vary Based on Identity Salience
Source: Pers Soc Psychol Bull. 2025 Jun 30;52(9):2709–23. doi: 10.1177/01461672251347735 (PMC13392178; doi:10.1177/01461672251347735)
Supplement: sj-docx-1-psp-10.1177_01461672251347735 – Supplemental material for The Comparative Status Hypothesis: Inferences About Discrimination Vary Based on Identity Salience [file sj-docx-1-psp-10.1177_01461672251347735.docx]

**The Comparative Status Hypothesis: Inferences about Discrimination Vary Based on Identity Salience:**

Supplementary Material

**Supplemental Materials Table of Contents**

| Contents | Page |
| --- | --- |
| Supplemental Materials Table of Contents | 2 |
| Studies 1-3: Internal Attribution Measures & Results | 3 |
| Study 1-3: Image Norming Ratings | 4-6 |
|  |  |

**Internal Attributions Measure & Results**

**Internal Attributions**. Three items measured internal attributions: “[Target]’s qualifications,” “[Target]’s career ambitions,” and “[Target]’s work record.” All ratings were assessed on a scale from 1 (“not at all”) to 7 (“very much”). Items were averaged to create a composite variable.

**Study 1 Results**

The ANOVA revealed no significant main effects of target race (*F*(1, 427) = 1.12, *p* = .292, η_р_^2^ = 0.00), competitor race (*F*(1, 427) = 1.84, *p* = .176, η_р_^2^ = 0.00), or interaction (*F*(1, 427) = 2.43, *p* = .120, η_р_^2^ = 0.01) on internal attributions for the funding outcome.

**Study 2 Results**

Competitor identity was not significantly related to participants’ ratings of whether the target lost out on the funding due to internal attributions (*F*(2, 587) = 1.86, *p* = .157, η_р_^2^ = 0.01).

**Study 3 Results**

Competitor identity did not significantly affect beliefs that the target lost out on the funding due to internal attributions (*F*(2, 586) = 1.16, *p* = .316, η_р_^2^ = 0.00).

**Study 1: Norming Data for Images Used Taken from Ma et al. (2015)**

| **Image #** | **Target/**  **Competitor** | **Prototypicality Rating** | **Age Rating** | **Attractiveness Rating** | **Trust**  **Rating** |
| --- | --- | --- | --- | --- | --- |
| AM-201 | Asian Male Target/Competitor | 4.55 | 23.80 | 3.04 | 3.12 |
| AM-210 | Asian Male Target/Competitor | 3.80 | 21.05 | 3.80 | 4.47 |
| AM-229 | Asian Male Target/Competitor | 3.48 | 24.76 | 3.48 | 3.19 |
| BM-019 | Black Male Target/Competitor | 4.15 | 21.61 | 3.87 | 3.99 |
| BM-200 | Black Male Target/Competitor | 4.22 | 26.48 | 3.22 | 4.04 |
| BM-029 | Black Male Target/Competitor | 4.04 | 29.84 | 3.00 | 3.54 |
| WM-003 | White Male Competitor | 4.19 | 23.35 | 3.68 | 3.58 |
| WM-026 | White Male Competitor | 4.06 | 25.68 | 3.09 | 3.17 |
| WM-214 | White Male Competitor | 4.13 | 25.12 | 3.12 | 3.72 |

**Study 2: Norming Data for Images Used Taken from Ma et al. (2015)**

| **Image #** | **Target/ Competitor** | **Prototypicality Rating** | **Age Rating** | **Attractiveness Rating** | **Trust Rating** |
| --- | --- | --- | --- | --- | --- |
| WF-001 | White Female Target | 4.27 | 24.95 | 3.11 | 3.30 |
| WF-005 | White Female Target | 4.21 | 22.39 | 3.03 | 3.75 |
| WF-231 | White Female Target | 4.56 | 26.31 | 3.86 | 3.34 |
| BF-037 | Black Female Competitor | 4.11 | 26.41 | 3.05 | 3.42 |
| BF-021 | Black Female Competitor | 3.96 | 27.93 | 3.38 | 3.25 |
| BF-003 | Black Female Competitor | 4.15 | 25.46 | 2.94 | 3.79 |
| WM-003 | White Male Competitor | 4.19 | 23.35 | 3.68 | 3.58 |
| WM-026 | White Male Competitor | 4.06 | 25.68 | 3.09 | 3.17 |
| WM-214 | White Male Competitor | 4.13 | 25.12 | 3.12 | 3.72 |

**Study 3: Norming Data for Images Used Taken from Ma et al. (2015)**

| **Image #** | **Target/ Competitor** | **Prototypicality Rating** | **Age Rating** | **Attractiveness Rating** | **Trust Rating** |
| --- | --- | --- | --- | --- | --- |
| AF-205 | Asian Female Target | 3.56 | 26.59 | 4.33 | 3.52 |
| AF-206 | Asian Female Target | 3.78 | 26.52 | 2.71 | 3.43 |
| AF-214 | Asian Female Target | 379 | 26.84 | 3.73 | 3.92 |
| BF-037 | Black Female Competitor | 4.11 | 26.41 | 3.05 | 3.42 |
| BF-021 | Black Female Competitor | 3.96 | 27.93 | 3.38 | 3.25 |
| BF-003 | Black Female Competitor | 4.15 | 25.46 | 2.94 | 3.79 |
| WF-001 | White Female Competitor | 4.27 | 24.95 | 3.11 | 3.30 |
| WF-005 | White Female Competitor | 4.21 | 22.39 | 3.03 | 3.75 |
| WF-231 | White Female Competitor | 4.56 | 26.31 | 3.86 | 3.34 |
| AM-201 | Asian Male Competitor | 4.55 | 23.80 | 3.04 | 3.12 |
| AM-210 | Asian Male Competitor | 4.58 | 21.05 | 3.80 | 4.47 |
| AM-229 | Asian Male Competitor | 4.19 | 24.76 | 3.48 | 3.19 |
